# Supplementary material for: Imaging non-collinear antiferromagnetic textures via single spin relaxometry
Source: Nat Commun. 2021 Feb 3;12:767. doi: 10.1038/s41467-021-20995-x (PMC7859235; doi:10.1038/s41467-021-20995-x)
Supplement: Supplementary file 1 — Supplementary Information [file 41467_2021_20995_MOESM1_ESM.pdf]

Supplementary Information
Imaging antiferromagnetic textures via single spin relaxometry

Aurore Finco,[1] Angela Haykal,[1] Rana Tanos,[1] Florentin Fabre,[1] Saddem Chouiaeb,[1] Waseem Akhtar,[1] Isabelle Robert-Philip,[1] William Legrand,[2] Fernando Ajejas,[2] Karim Bouzehouane,[2] Nicolas Reyren,[2] Thibaut Devolder,[3] Jean-Paul Adam,[3] Joo-Von Kim,[3] Vincent Cros,[2] and Vincent Jacques[1]

[1]*Laboratoire Charles Coulomb, Université de Montpellier and CNRS, 34095 Montpellier, France*
[2]*Unité Mixte de Physique, CNRS, Thales, Université Paris-Saclay, 91767 Palaiseau, France*
[3]*Centre de Nanosciences et de Nanotechnologies, CNRS, Université Paris-Saclay, 91120 Palaiseau, France*

CONTENTS

Supplementary Note 1. Experimental details 2
1.1. Magnetic characterization of the SAF samples 2
1.2. Scanning-NV magnetometry setup 2
1.3. Characterization of the scanning-NV sensor 3

Supplementary Note 2. Calculation of the PL dependence on T_1 5
2.1. Two-level model 5
2.2. Qualitative comparison with pulsed-laser relaxometry 5

Supplementary Note 3. Micromagnetic simulations 7
3.1. Micromagnetic parameters 7
3.2. Principle of the calculation of the noise maps 7
3.3. Simulated dispersion relation for spin spirals 9
3.4. Simulated noise spectrum of an isolated SAF skyrmion 10
3.5. Thermal power spectral density of the SAF uniform resonance mode 11

Supplementary Note 4. Additional experimental data 13
4.1. Quantitative analysis of the domain wall profile 13
4.2. Measurements of T_1 above non-magnetic surfaces 13
4.3. Additional relaxation time measurements on the spin spiral sample 14
4.4. Additional relaxation time measurements on the skyrmion sample 16

Supplementary Note 1. EXPERIMENTAL DETAILS

1.1. Magnetic characterization of the SAF samples

The magnetic multilayers have been deposited by d.c. magnetron sputtering at room temperature on thermally oxidized silicon substrates, under Ar gas flow at a pressure of 0.25 Pa with deposition rates calibrated beforehand by x-ray reflectivity measurements. The base pressure of the sputtering equipment was 5×10^{-6} Pa. All multilayers described in this work have been deposited on top of Ta(5 nm)/Pt(8 nm) buffers and capped with Pt(3 nm) layers to prevent oxidation.

A preliminary characterization of the sample magnetization have been performed using an alternating gradient magnetometry setup, on 4 mm × 4 mm films. The out-of-plane magnetization hysteresis loops of the multilayer SAFs imaged in Figs. 2 and 5 are presented in Supplementary Fig. 1. They consist of the average out-of-plane magnetization component m_z summed over all magnetic layers, which is measured as a function of the externally applied out-of-plane magnetic field $\mu_0 H_z$. The loop for the SAF with $t_{\rm Co} = 1.41$ nm shows a rather flat central plateau at zero magnetization ended by transitions toward saturated magnetization states at its two ends, indicative of a significant effective magnetic anisotropy (Supplementary Fig. 1**a**). The SAF with $t_{\rm Co} = 1.47$ m rather shows a linear evolution of its average m_z towards saturation (Supplementary Fig. 1**b**), here characteristic of a vanishing effective anisotropy allowing for the stabilization of a spin-spiral state under zero external field. For the biased SAF, this loop is superimposed to the one of the bias layer, which appears in Supplementary Fig. 1**c** as the central rectangular sharp loop. The remaining of the curve features the evolution of m_z in the SAF system coupled to it, with the same linear evolution, as $t_{\rm Co} = 1.47$ nm. These curves also reveal that the SAF system hosts a compensated magnetic configuration at zero external field, in all three cases.

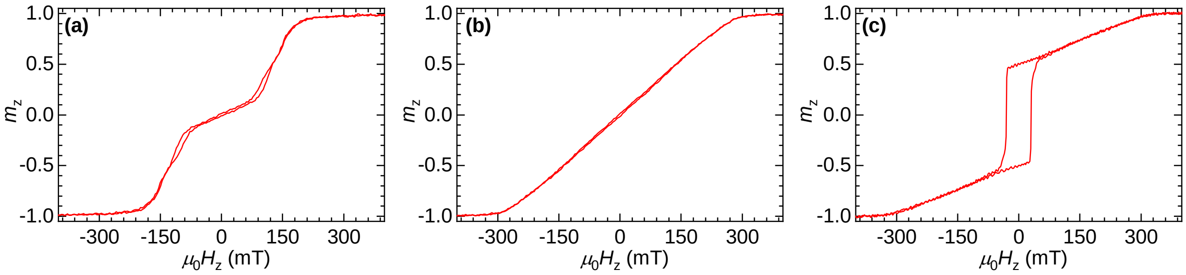

Supplementary Figure 1. **Hysteresis loops.** Average out-of-plane magnetization component m_z of the full stacks for **a** the domain wall sample ($t_{\rm Co} = 1.41$ nm), **b** the spiral sample ($t_{\rm Co} = 1.47$ nm) and **c** the skyrmion sample ($t_{\rm Co} = 1.47$ nm with an additional bias layer).

1.2. Scanning-NV magnetometry setup

The experimental setup is described in details in Ref. [1]. It combines a tuning-fork-based atomic force microscope (AFM) and a confocal optical microscope, all operating under ambient conditions. In this work, a commercial diamond tip (Qnami, Quantilever MX) hosting a single NV defect at its apex is attached to the AFM head and scanned above

the magnetic sample. At each point of the scan, a confocal optical microscope placed above the tip is used to monitor the spin-dependent PL of the NV defect under green laser illumination. The diameter of the scanning diamond tip is around 200 nm in order to act as an efficient waveguide for the PL emission of the NV defect [2, 3]. Experiment control and data acquisition are carried out by Python programs from the collaborative Qudi project [4].

1.3. Characterization of the scanning-NV sensor

The distance d_{NV} between the scanning-NV spin sensor and the sample surface was independently calibrated by recording the stray magnetic field produced above the edges of an uniformly magnetized ferromagnetic wire, following the procedure detailed in Ref. [5]. A typical Zeeman-shift profile recorded while scanning the NV defect across the edges of a 1 µm-wide wire of Pt/Co(0.6 nm)/AlO$_x$ is shown in Supplementary Fig. 2**a**. The distance d_{NV} is then extracted by fitting the experimental data (red dashed line in Supplementary Fig. 2**a**). For the scanning diamond tip used in this work, we obtain $d_{\mathrm{NV}} = 79 \pm 5$ nm. The analysis of uncertainties is discussed in Ref. [5].

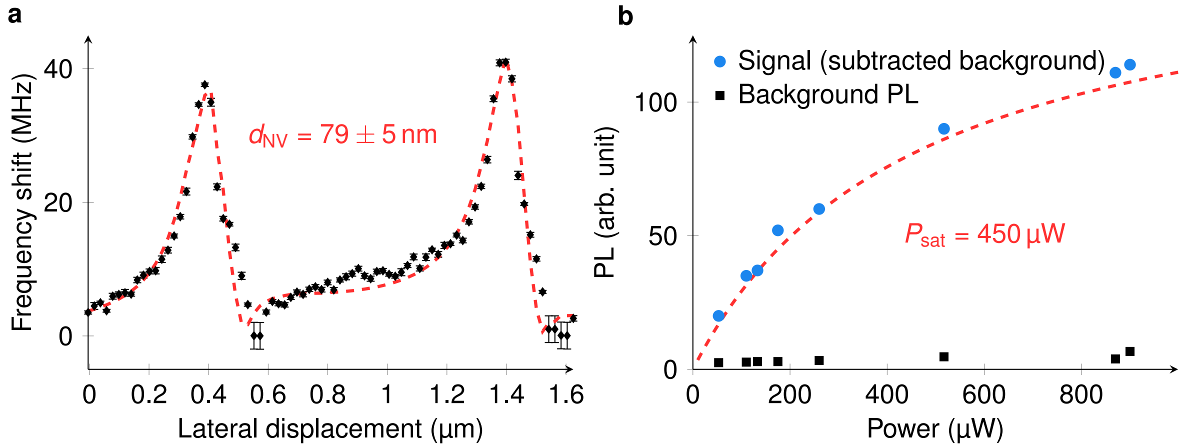

Supplementary Figure 2. **Characterization of the scanning-NV sensor**. **a** Stray field profile recorded across the edges of a 1 µm-wide wire of Pt/Co(0.6nm)/AlO$_x$ with perpendicular magnetic anisotropy. The markers are experimental data and the red dashed line is data fitting, from which the NV-to-sample distance d_{NV} is extracted. We note that only the absolute value of the magnetic field is measured in this experiment. **b** Saturation curve of the NV defect PL signal with the tip engaged on the sample surface. The red dashed line is a data fitting with a saturation function, leading to $P_{\mathrm{sat}} = 450$ µW. The signal to background ratio is around 10 at low excitation power and reaches values above 20 close to saturation.

The NV defect quantization axis was measured by recording the electron spin resonance (ESR) frequency as a function of the amplitude and orientation of a calibrated magnetic field, leading to spherical angles $\theta_{\mathrm{NV}} = 58°$ and $\phi_{\mathrm{NV}} = 103°$ in the laboratory frame of reference (x, y, z).

Another important parameter of the NV sensor is the saturation power P_{sat} of its optical transition. This parameter was estimated by recording the PL rate $\mathcal{R}$ as a function of the optical illumination power P, with the tip engaged above the sample surface (see Supple-

mentary Fig. 2**b**). Data fitting with a simple saturation function $\mathcal{R} \propto \frac{P}{P + P_{\text{sat}}}$ leads to a saturation power $P_{\text{sat}} = 450\,\mu\text{W}$. We note that background PL signal emanating from the tip and/or the sample remains very weak in the whole range of optical powers used in this work.

Supplementary Note 2. CALCULATION OF THE PL DEPENDENCE ON T_1

2.1. Two-level model

We consider the simple two-level system presented in Fig. 1**a** to infer the evolution of the NV defect PL signal with T_1. Under continuous optical illumination with a power P, the steady state spin populations $(n_0^{\rm st}, n_1^{\rm st})$ result from the competition between the longitudinal spin relaxation rate $\Gamma_1 = \frac{1}{2T_1}$, and optically-induced spin polarization in the $m_s = 0$ spin sublevel with a rate Γ_p, which can be expressed as

$$\Gamma_p = \Gamma_p^{\infty} \frac{P}{P + P_{\rm sat}} \ ,$$

where $\Gamma_p^{\infty} \simeq 5 \times 10^6\,{\rm s}^{-1}$ denotes the polarization rate at saturation [6]. Using this simple model, the steady state spin populations under continuous optical illumination are given by

$$n_0^{\rm st} = \frac{\Gamma_1 + \Gamma_p}{2\Gamma_1 + \Gamma_p} \ , \ n_0^{\rm st} + n_1^{\rm st} = 1 \ . \tag{1}$$

The PL rate $\mathcal{R}$ of the NV defect can then be expressed as

$$\mathcal{R} = \frac{P}{P + P_{\rm sat}} \left(\mathcal{R}_0^{\infty} n_0^{\rm st} + \mathcal{R}_1^{\infty} n_1^{\rm st} \right) \ , \tag{2}$$

where $\mathcal{R}_0^{\infty}$ (resp. $\mathcal{R}_1^{\infty}$) denotes the saturated PL rate resulting from the population in the $m_s = 0$ (resp. $m_s = \pm 1$) spin sublevel. The spin-dependent PL response of the NV defect is phenomenologically introduced by considering that $\mathcal{R}_1^{\infty} = \beta \mathcal{R}_0^{\infty}$, where $\beta < 1$ [6]. The PL rate then writes

$$\mathcal{R} = \mathcal{R}_0^{\infty} \frac{P}{P + P_{\rm sat}} \frac{(1+\beta)\Gamma_1 + \Gamma_p}{2\Gamma_1 + \Gamma_p} \ . \tag{3}$$

In the limit $\Gamma_1 \ll \Gamma_p$, the NV defect is efficiently polarized in the $m_s = 0$ spin sublevel ($n_0^{\rm st} \sim 1$), leading to a maximal PL rate $\mathcal{R}_{\rm max} = \mathcal{R}_0^{\infty} \frac{P}{P + P_{\rm sat}}$.

The curves shown in Fig. 1**a** correspond to the ratio $\mathcal{R}/\mathcal{R}_{\rm max}$ calculated as a function of T_1 for two different optical excitation power while using $\beta = 0.6$.

2.2. Qualitative comparison with pulsed-laser relaxometry

As indicated by the main manuscript, the laser power needs to be properly adjusted to optimize the imaging contrast. More precisely, the NV sensor is less sensitive when used to image weak magnetic noise since the optical illumination power has to be significantly decreased, resulting in a lower PL signal. Another way to record variations of the NV spin relaxation time consists in recording the PL signal while applying laser pulses separated by a fixed time delay τ. This method, commonly referred to as single-τ imaging mode, was used in the past to image paramagnetic nanoparticles [7–9]. In a single-τ imaging procedure, this is the delay time τ between laser pulse excitations which needs to be properly adjusted

in order to optimize the signal to noise ratio. For the detection of a weak magnetic noise, this delay has to be long, typically on the order of T_1 [10]. As a consequence, the duty cycle of optical excitation needs to be reduced, resulting in a decreased PL signal by a factor T_L/T_1, where T_L is the duration of the laser pulses. To optimize spin readout contrast, the laser pulse duration is commonly set to $T_L = 300\,\text{ns}$ with a power close to the saturation power (P_{sat}) of the optical transition [6]. Considering $T_1 = 100\,\mu\text{s}$, the PL signal is thus decreased by two orders of magnitude compared to continuous laser illumination. Using our measurement procedure, the cw laser power has to be decreased to $0.01 \times P_{\text{sat}}$ in order to be sensitive to weak magnetic noise (see Fig. 1**b**) leading to similar PL signals. As a result, the two approaches are likely to offer similar performances. An in-depth analysis of the magnetic noise sensitivity would require a precise study of (i) the PL quenching effect with a calibrated magnetic noise source and (ii) its evolution with the optical pumping power. This will likely require to introduce more sophisticated models of the NV defect's photodynamics than the one introduced in our work.

Supplementary Note 3. MICROMAGNETIC SIMULATIONS

3.1. Micromagnetic parameters

The magnetic noise spectrum is investigated using numerical micromagnetics with the MuMax3 code [11, 12]. The SAF structure was modelled as a rectangular trilayer system with dimensions of L_x nm × L_y nm × 4.5 nm that is discretised with $N_x \times N_y \times 3$ finite difference cells. According to experimental measurements on the samples, we use a saturation magnetization of $M_s = 1.2\,\mathrm{MA\,m^{-1}}$, a ferromagnetic intralayer exchange of $A = 20\,\mathrm{pJ\,m^{-1}}$, and a uniaxial magnetic anisotropy along the z axis, perpendicular to the film plane. For the domain wall system, we consider a uniaxial anisotropy constant of $K_u = 0.945\,\mathrm{MJ\,m^{-3}}$, an interfacial Dzyaloshinskii-Moriya constant of $D = 0.9\,\mathrm{mJ\,m^{-2}}$, and an antiferromagnetic interlayer exchange coupling of $A_{\mathrm{RKKY}} = -0.3\,\mathrm{mJ\,m^{-2}}$, while for spin spiral and skyrmion system we consider $K_u = 0.895\,\mathrm{MJ\,m^{-3}}$ (leading to a nearly vanishing $K_{\mathrm{eff}} = K_u - \mu_0 M_s^2/2$) , $D = 0.85\,\mathrm{mJ\,m^{-2}}$, and $A_{\mathrm{RKKY}} = -0.27\,\mathrm{mJ\,m^{-2}}$. For the skyrmion system, an additional bias field of $\mu_0 H_b = 50\,\mathrm{mT}$ is applied to the bottom layer of the SAF, which mimics an exchange bias coupling. For the domain wall system, periodic boundary conditions are applied along the y direction, parallel to the length of the domain wall, while for the spin spiral and skyrmion systems periodic boundaries are applied in both the x and y directions. A Gilbert damping constant of $\alpha = 0.1$ was used for all systems.

The dispersion relations in Fig. 4**b-c** were constructed from the transient response to a time-dependent magnetic field of the form $\mathbf{b}_{\mathrm{rf}}(y,t) = b_0(t)\,[\cos(k_n y)\hat{\mathbf{x}} + \sin(k_n y)\hat{\mathbf{y}}]$ for the uniform state and $\mathbf{b}_{\mathrm{rf}}(y,t) = b_0(t)\,[\cos(k_n y)\hat{\mathbf{x}} + \sin(k_n y)\hat{\mathbf{z}}]$ for the domain wall and spin spiral, where $b_0(t) = \sin(\pi\nu t)/\pi\nu t$ is a sinc function with $\nu = 50\,\mathrm{GHz}$ and amplitude of 1 mT, while $k_n = 2\pi n/N_y$ is a quantized wave vector along the propagation direction, y. For all simulations we used $N_y = 2048$, $L_y = 10\,240\,\mathrm{nm}$, while $N_x = 64$, $L_x = 160\,\mathrm{nm}$ for the uniform state, $N_x = 256$, $L_x = 640\,\mathrm{nm}$ for the domain wall, and $N_x = 128$, $L_x = 250\,\mathrm{nm}$ for the spin spiral configuration. For each value of the wave vector k_n, $b_{\mathrm{rf}}(t)$ was applied to the system starting from the initial relaxed magnetic configuration and the damped magnetic response was simulated over 20 ns. The power spectrum was then computed from the Fourier transform of this magnetic response. Let $l = 1, 2$ denote the top and bottom layers of the SAF, respectively. In Fig. 4, we present the dispersion relations corresponding to the sum response, $\mathbf{m}_+ = (\mathbf{m}_1 + \mathbf{m}_2)/2$, which represents the net magnetization that would be probed experimentally.

3.2. Principle of the calculation of the noise maps

To estimate the relevant magnetic noise spectrum for the relaxometry measurements, we computed the response of the SAF to a random spatial field which simulates thermal fluctuations, but with a harmonic time dependence at the frequency of $f_0 = 2.87\,\mathrm{GHz}$. The idea is to estimate the power spectrum of magnetic fluctuations at f_0 by computing the magnetic susceptibility at this frequency. This random field varies between each finite difference cell, where its orientation is drawn from a uniform distribution over the unit sphere (i.e., the z component is taken to be a random value between $[1, -1]$ and the azimuthal angle is drawn randomly from the interval $[0, 2\pi]$, while its magnitude is drawn from a Gaussian distribution centred at zero with a variance of 1 mT (see Supplementary Fig. 3**a**). The random field is applied to a relaxed magnetic state and the simulation geometry for all cases

is $N_x = N_y = 512$, $L_x = L_y = 750\,\text{nm}$.

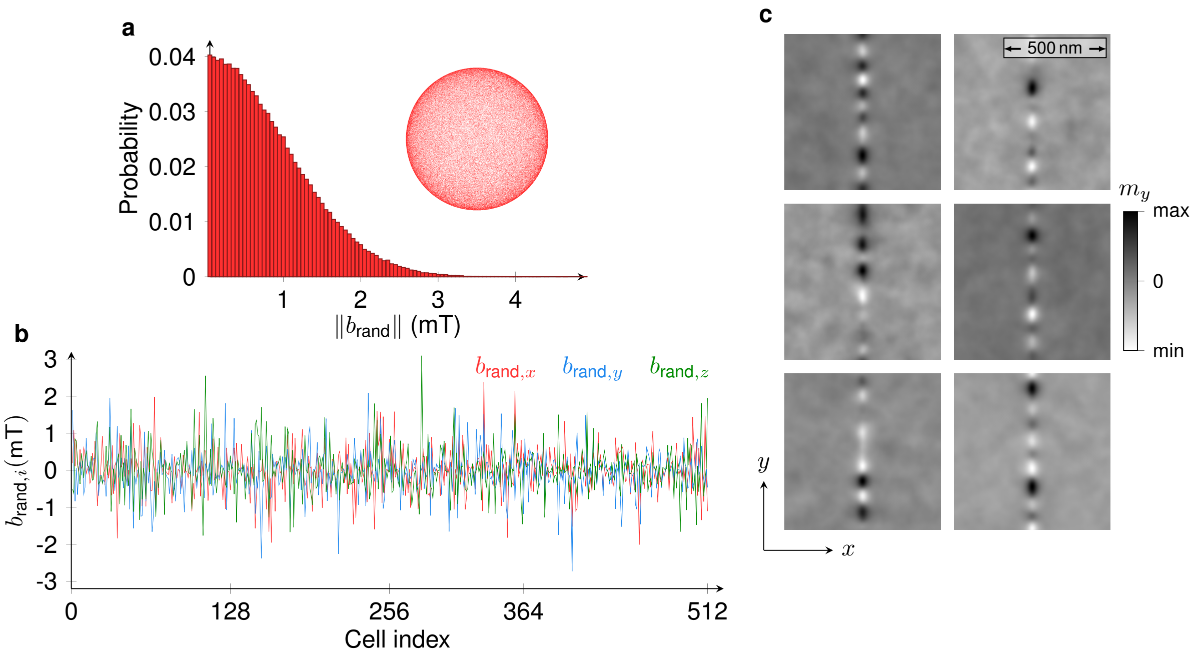

Supplementary Figure 3. **Random field excitation for noise intensity maps.** **a** Probability distribution of field magnitude for one realisation of the spatial random field, $\mathbf{b}_{\text{rand}}(\mathbf{r}, t)$. The inset shows the field orientations projected onto the unit sphere. **b** Example of a line cut of one realisation of the random field across the width of the simulation grid. **c** Six snapshots of the m_y response in the bottom SAF layer to different realizations of the random spatial field with a sinusoidal time dependence at a frequency of 2.87 GHz.

The noise map is computed as follows. For a given realisation i of the random magnetic field, the dynamics is calculated using the MuMax3 code over one period of the 2.87 GHz excitation and the micromagnetic state is recorded. We then compute the deviation in the stray magnetic field with respect to the relaxed state $\mathbf{m}_0$, $\delta\mathbf{B}_i = \mathbf{B}_i - \mathbf{B}_0$, at a height of 80 nm above the multilayer, which corresponds to the flying distance of the NV spin sensor. The square of the magnitude of the projection of this field along the direction perpendicular to the NV axis, $||\delta\mathbf{B}_{\perp,i}||^2$, is then recorded at each position of the simulation grid. This projection operation is motivated by the fact that the longitudinal spin relaxation time of the NV defect is only sensitive to the magnetic noise component perpendicular to the NV axis [13]. The noise maps shown in Fig. 4**d**, Supplementary Fig. 4**d,f** and Supplementary Fig. 5**d,f** represent an average of $||\delta\mathbf{B}_{\perp,i}||^2$ over 500 different realizations of the random driving field.

Magnetic disorder was included to obtain more realistic static magnetization profiles, such as roughening of domain walls and spin spirals, and non axially-symmetric skyrmions. Disorder was simulated by local fluctuations in the uniaxial anisotropy constant. By using Voronoi tessellation, we divided the bilayer into a distribution of grains [12] in the film plane with an average grain size of 10 nm. The anisotropy constant in each grain j, $K_{u,j}$, was drawn from a Gaussian distribution centred on the base value $K_{u,0}$ used previously with a spread of 5%. The ground state magnetization was computed by relaxing the equilibrium profile computed in the disorder-free cases in the presence of these grains.

3.3. Simulated dispersion relation for spin spirals

With the micromagnetic parameters given at the beginning of Supplementary Note 3, we first initialize the system with a sinusoidal magnetization state with a wave vector along the x direction and the magnetic moments rotating in the xz-plane. To model the experimental system, the initial state comprises one full period over the size of the supercell of 250 nm, with periodic conditions applied along both the x and y directions. The moments in the top and bottom layers of the SAF are aligned in antiparallel with a left-handed chirality, as appropriate for the sign of the Dzyaloshinskii-Moriya interaction chosen. This initial trial state is then relaxed toward an equilibrium state by minimizing the energy. The relaxed configuration is shown in Supplementary Figure 4**a**.

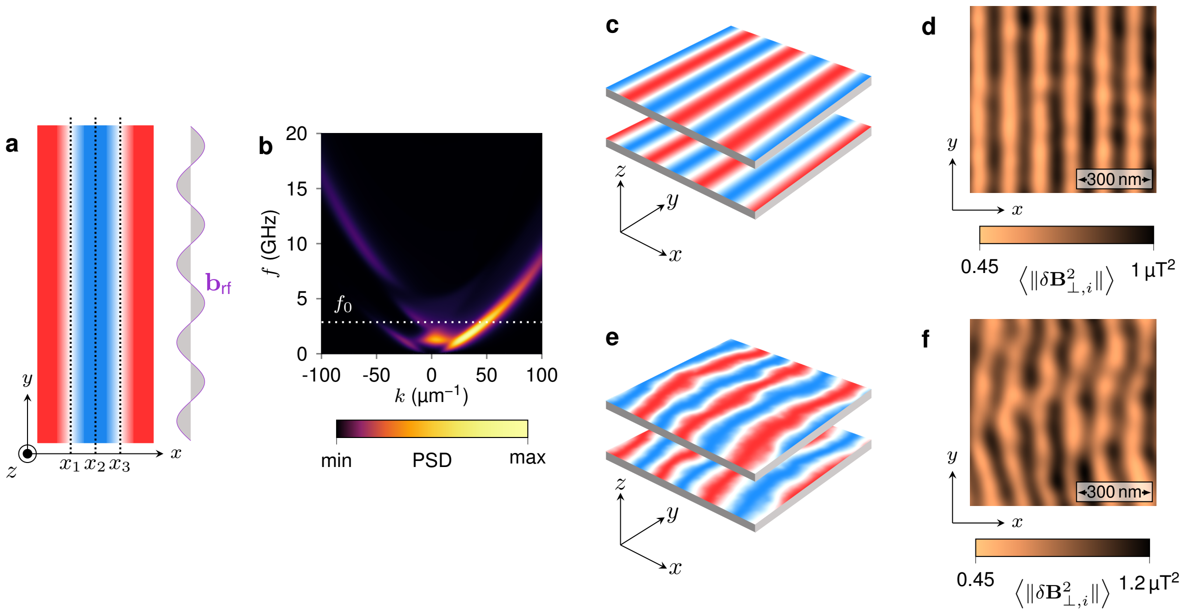

Supplementary Figure 4. **Spin wave dispersion relations and noise maps in SAF spin spirals.** **a** Relaxed spin spiral configuration used for the calculation of the dispersion relation. The vertical dashed lines indicate the positions ($x = x_1, x_2, x_3$) at which the the spin wave response is computed, and b_{rf} denotes a schematic of the spatial sinusoidal field applied. **b** Combined dispersion relations obtained at $x = x_1, x_2, x_3$ in the geometry given in **b**, where the dashed line indicates the NV frequency, $f_0 = 2.87$ GHz. **c** Relaxed spin spiral configuration used for magnetic noise intensity simulations. **d** Simulated magnon noise map at $f_0 = 2.87$ GHz for the configuration in **d** at 80 nm from the surface. **e** Relaxed spin spiral configuration in the presence of magnetic disorder. **f** Simulated magnon noise map at f_0 for the configuration in **e** at 80 nm from the surface.

To compute the spin wave dispersion relations in the spin spiral, we follow the method used for the domain wall (Fig. 4**c** in the main text). We consider the transient response of the configuration in Supplementary Fig. 4**a** to a spatial sinusoidal field of different wave vectors, which are quantised by the length of the simulated geometry, with a sinc time dependence as discussed previously. The response is recorded at three positions along x: at the two channels along which $m_z = 0$ (at $x = x_1$ and $x = x_3$ in Supplementary Fig. 4**a**) and at $m_z = \pm 1$ (at $x = x_2$ in Supplementary Fig. 4**a**). Because of the proximity of these three positions in the spin spiral, there is some intermixing between the modes and no clear

distinction between “bulk” and “channelled” as in the domain wall case. The simulated dispersion relations are shown in Supplementary Fig. 4**b**.

In Supplementary Fig. 4**c**, we show the relaxed spin spiral configuration for the calculation of the noise map. The geometry simulated is $750 \times 750 \times 4.5\,\mathrm{nm}^3$ in size that is discretized with $512 \times 512 \times 3$ finite difference cells. The simulated noise map at 2.87 GHz is shown in Supplementary Fig. 4**d**, where we can observe a clear spatial contrast corresponding to the spin spiral configuration. The relaxed configuration in the presence of magnetic disorder is given in Supplementary Fig. 4**e**, where we can observe a roughening of the spirals. The corresponding noise map of the disordered system is presented in Supplementary Fig. 4**f**. Again, a clear contrast of the spin spiral can be observed which is strongly correlated with the shape of the equilibrium magnetic profile.

3.4. Simulated noise spectrum of an isolated SAF skyrmion

To simulate the noise spectrum of an isolated SAF skyrmion, we used the same micromagnetic parameters as those for the spin spiral configuration with an additional static bias magnetic field, $\mu_0 H_b = 50\,\mathrm{mT}$, which is applied to the bottom layer of the SAF. Since the effective perpendicular magnetic anisotropy is vanishing in this case, the presence of this bias field helps to stabilise the perpendicular magnetization. In Supplementary Fig. 5**a**, we plot the dispersion relation of the propagating spin waves in the uniform state with these parameters. These curves were obtained by linearizing the Landau-Lifshitz equation for the coupled dynamics of the top and bottom layer, using thin film approximations for the dipolar interactions [14]. In contrast to the case shown in Fig. 4**b** of the main text, where the spin wave gap scales with the perpendicular magnetic anisotropy, here the gap is largely dominated by the bias field applied to the bottom layer of the SAF, in conjunction with the interlayer exchange coupling, which also results in a larger frequency splitting between the two branches. The lower frequency branch, ‘t’, involves a stronger mode localization to the top magnetic layer in which the bias field is absent, while the higher frequency branch, ‘b’, involves stronger mode localization to the bottom magnetic layer in which the bias field is present. Another key difference compared with the spin wave spectrum in the uniform domains in Fig. 4 is the presence of excitation modes at the NV frequency. The inclusion of Gilbert damping results in the smeared PSD in Supplementary Fig. 5**b**, which shows a colour map computed from micromagnetics simulations.

The static magnetization profiles of the skyrmion in the top and bottom layers are shown in Supplementary Fig. 5**c**. The geometry simulated is $750 \times 750 \times 4.5\,\mathrm{nm}^3$ in size that is discretized with $512 \times 512 \times 3$ finite difference cells. The simulated noise intensity map is shown in Supplementary Fig. 5**d**. Like for the domain wall and spin spiral cases, this map represents an average over 500 realisations of a random field with a harmonic time dependence at a frequency of 2.87 GHz. The map represents the values of the noise taken at 80 nm above the skyrmion. Despite the fact that the internal breathing mode frequencies of the skyrmion is above 2.87 GHz, we can observe a strong noise contrast at this frequency. We attribute this to the scattering of propagating spin waves with the skyrmion, which results in small displacements and deformations of the equilibrium profile. When disorder is included, we obtain the deformed skyrmion core as shown in the equilibrium profile in Supplementary Fig. 5**e**. The simulated noise map in Supplementary Fig. 5**f** reveals a smeared out contrast, both in the proximity of the skyrmion and within the domain, which arises from random scattering of the propagating spin waves at the NV frequency.

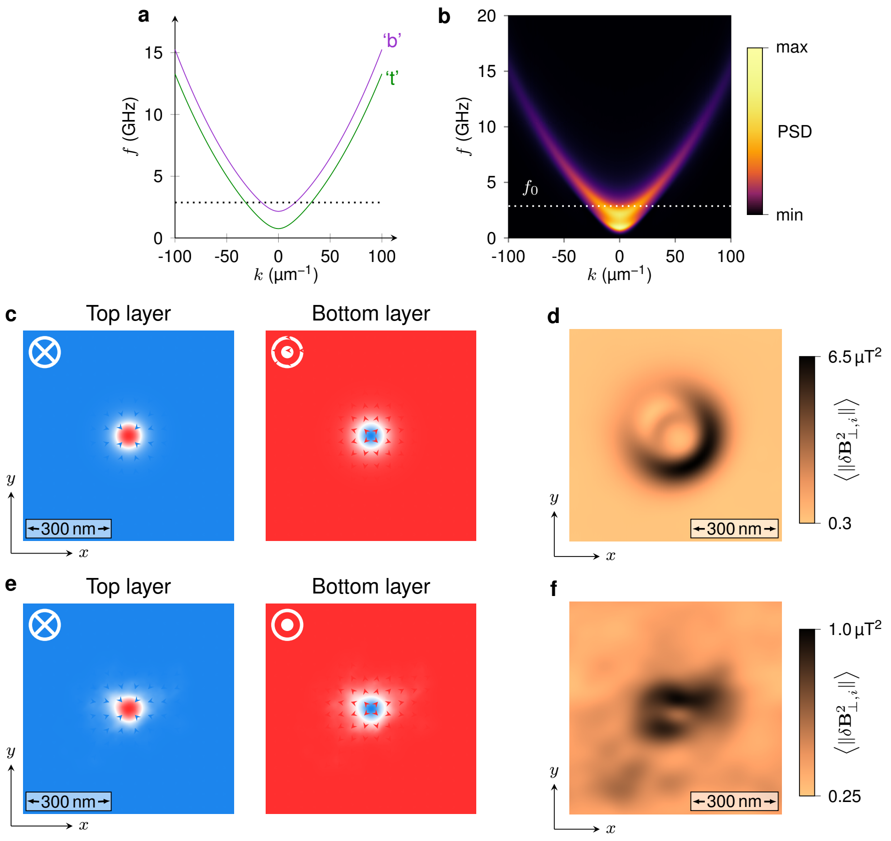

Supplementary Figure 5. **Noise spectrum of an isolated SAF skyrmion.** **a** Theoretical dispersion relation for the uniform SAF domain with biasing on the bottom layer. 'b' and 't' denote strong mode localization in the bottom and top layers of the SAF, respectively. **b** Simulated dispersion relation for the domain state with $\alpha = 0.1$. The dashed lines in **a** and **b** indicate the NV frequency, $f_0 = 2.87\,\mathrm{GHz}$. **c** Relaxed magnetic configuration of the skyrmion. **d** Simulated map of the magnetic noise intensity at f_0 for the configuration in **c** at 80 nm from the surface. **e** Relaxed magnetic configuration of the skyrmion in the presence of magnetic disorder. **f** Simulated map of the magnetic noise intensity at f_0 for the configuration in **e** at 80 nm from the surface.

3.5. Thermal power spectral density of the SAF uniform resonance mode

In the uniform state and in the absence of applied magnetic fields, the spin wave gap is determined by the anisotropy constant and interlayer exchange coupling, and corresponds to the precession frequency of the uniform resonance mode of the antiferromagnetically-coupled bilayer. While this gap is well above the NV frequency of 2.87 GHz at zero temperature, the presence of a large Gilbert damping constant of $\alpha = 0.1$ results in a broadening of the spectral line such that the tail of the resonance peak overlaps the frequency range in which the NV spin relaxation is important. An example of the power spectrum is shown in Supplementary Fig. 6, where the spectral line at $T = 0\,\mathrm{K}$ was computed from the Fourier transform of the transient response of $\mathbf{m}_+(t) = \mathbf{m}_1(t) + \mathbf{m}_2(t)$ to the pulsed fields used to construct Fig. 4**b** of the main text. Indeed, this PSD represents the $k = 0$ point in Fig. 4**b**.

To examine how this PSD evolves with temperature, we performed additional micromagnetics simulations with the MuMax3 code [11] that incorporate thermal effects through

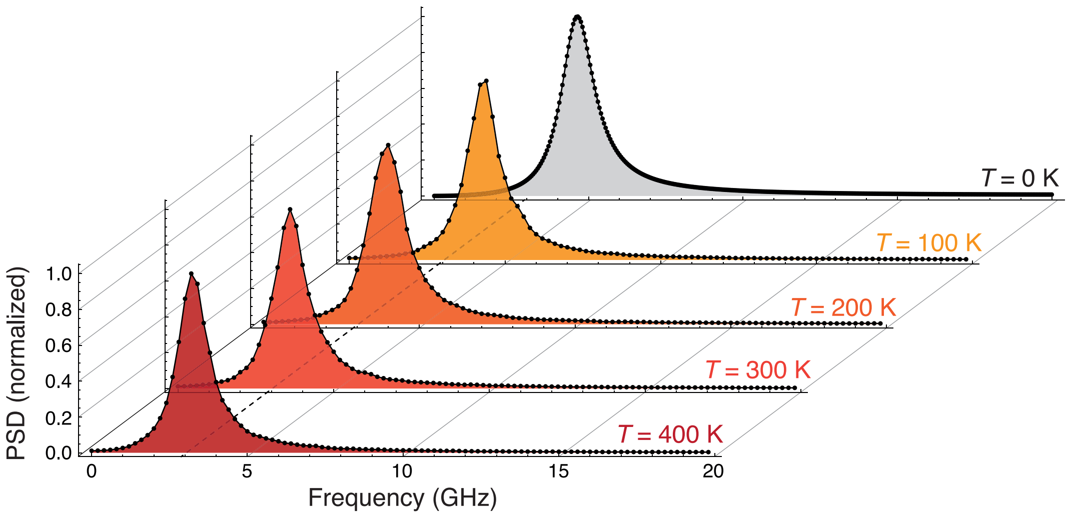

Supplementary Figure 6. **Thermal power spectral density (PSD) of the SAF uniform resonance mode.** At $T = 0\,\mathrm{K}$, the PSD was computed from the transient response to a sinc field. At finite temperatures, the PSD was computed from the stochastic magnetization dynamics over $2\,\mu\mathrm{s}$. The dashed line indicates the NV frequency of 2.87 GHz.

the inclusion of a random thermal field [15–17]. For each value of the temperature considered, we perform the time integration of the stochastic dynamics over an interval of $2\,\mu\mathrm{s}$. The power spectrum of $\mathbf{m}_+(t)$ is then obtained by using the Welch method, which involves averaging over the spectra generated from the Fourier transform of half-overlapping 5-ns windows into which the original time series data is sliced. The thermal spectra are shown in Supplementary Fig. 6 for several values of the temperature. We observe that the uniform resonance frequency exhibits a noticeable downward frequency shift, from 4.6 GHz at $T = 0\,\mathrm{K}$ to 3.6 GHz at $T = 300\,\mathrm{K}$. As such, it is likely that the relaxation broadening of this peak contributes to the observable reduction in T_1 over uniform domains.

Supplementary Note 4. ADDITIONAL EXPERIMENTAL DATA

4.1. Quantitative analysis of the domain wall profile

We compare the stray field profile of the domain wall presented in Figure 2 of the main text and again in Supplementary Fig. 7**a** with the profile expected from micromagnetic simulations. The magnetization profile of the domain wall plotted in Supplementary Fig. 7**b-c** is obtained from micromagnetic simulations with the parameters given in Supplementary Note 3. Because of the dipolar coupling between the two layers, the wall width is larger in the top layer ($w_{\text{top}} = 24\,\text{nm}$) than in the bottom layer ($w_{\text{bottom}} = 19.1\,\text{nm}$). The stray field which is probed by the NV center above the surface of the SAF results from this width difference and from the vertical spacing between the two layers. As shown in Supplementary Fig. 7**d**, we computed the projection of the stray field along the NV axis, which is the sum of the contributions of each layer. The solid line is the profile at a distance $d_{\text{NV}} = 79\,\text{nm}$ from the surface and the colored area corresponds to a variation of $\pm 5\,\text{nm}$. The width of the measured peak is thinner than calculated, but this can be explained by drift during the scan because the domain wall is parallel to the fast scanning direction. We obtain a qualitative agreement with the experimental data in terms of field amplitude.

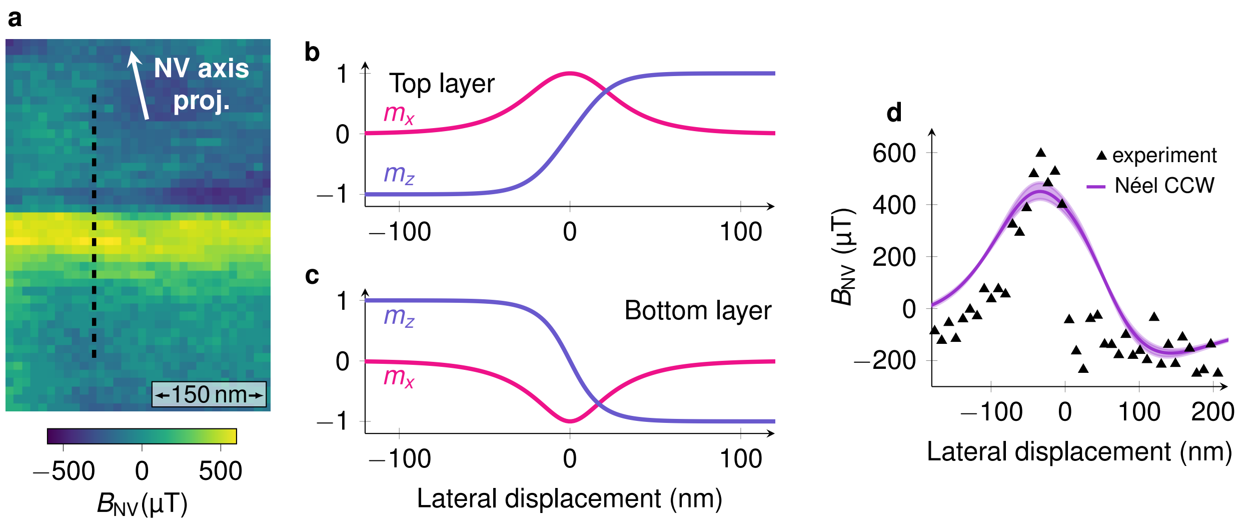

Supplementary Figure 7. **Analysis of the domain wall profile.** **a** Field map recorded above a domain wall in a SAF sample, showing the component of the stray field oriented along the NV axis. **b-c** Magnetization profiles in the top (**b**) and bottom (**c**) layers from micromagnetic simulation. **d** Comparison between the experimental field profile and simulations of the expected stray field for a Néel wall with a counterclockwise rotational sense.

4.2. Measurements of T_1 above non-magnetic surfaces

In order to check that the shortening of the T_1 time observed when the NV center is placed above a magnetic domain has a magnetic origin (Fig. 3**e**), we performed similar experiments on non-magnetic samples. Supplementary Fig. 8**a** shows a relaxation curve with the tip far

away from any surface, whereas the plots **b** and **c** display curves measured with the tip engaged on a Pt film or on a bulk diamond surface.

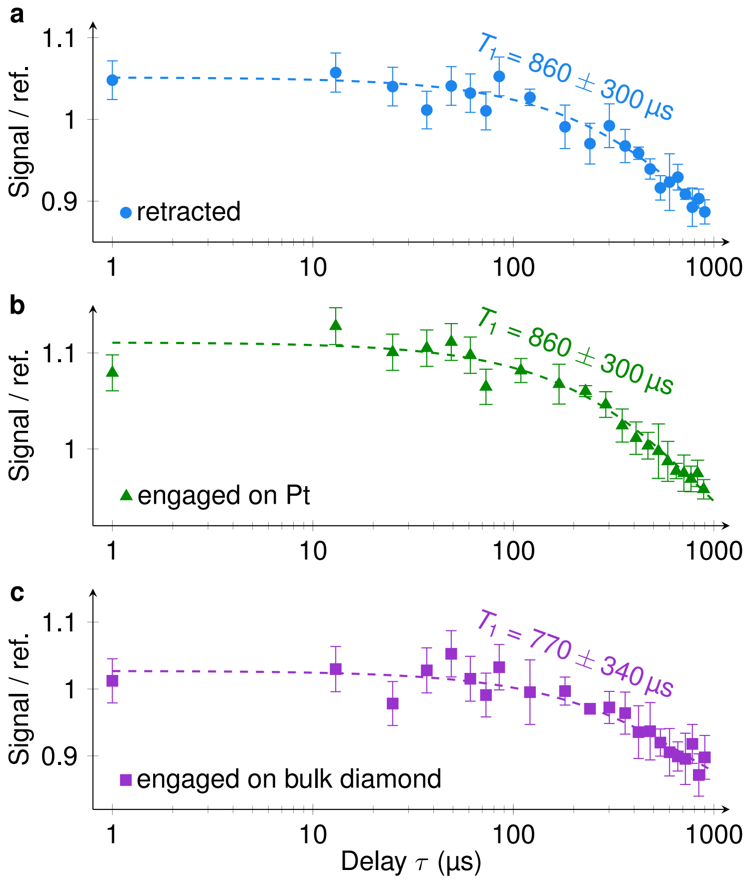

Supplementary Figure 8. **Relaxation time above non-magnetic surfaces**. T_1 measurements comparing the situations with **a** the tip retracted and **b** engaged on a Pt film or **c** bulk diamond.

We do not observe any significant variations of T_1 between these three cases. If the enhancement of the spin relaxation rate observed above a SAF domain was related to Johnson noise from the Pt capping layer of our magnetic samples, we should see a clear decrease of T_1 when we approach the tip to a pure Pt layer. Our control experiments allows to exclude this hypothesis. Shortening of T_1 could also originate from charge state conversion of the NV center when it is brought very close to a surface, but again, if it was the case this should also occur during our control experiments. These measurements therefore indicate that the decrease of T_1 measured when the tip is engaged on a SAF domain results from the presence of thermal population of magnons. This interpretation is further confirmed by the dispersion relation displayed in Fig. 4**c** of the main text and by the discussion about the effect of finite temperature in Supplementary Note 3 3.5.

4.3. Additional relaxation time measurements on the spin spiral sample

We performed measurements of the NV spin relaxation curve on the sample hosting the spin spiral, above the bright and the dark PL areas, as shown in Supplementary Fig. 9.

T_1 is smaller above the dark PL areas ($T_1 = 400 \pm 40\,\mathrm{ns}$) than above the bright PL areas ($T_1 = 920 \pm 60\,\mathrm{ns}$). The obtained values are much shorter than the one obtained on the sample hosting isolated domain walls. This is expected from the dispersion relation presented in Supplementary Fig. 4**b**, with a very small gap and strong modes at $f_0 = 2.87\,\mathrm{GHz}$. The detectable magnetic noise which they produce is more intense that in the domain wall sample.

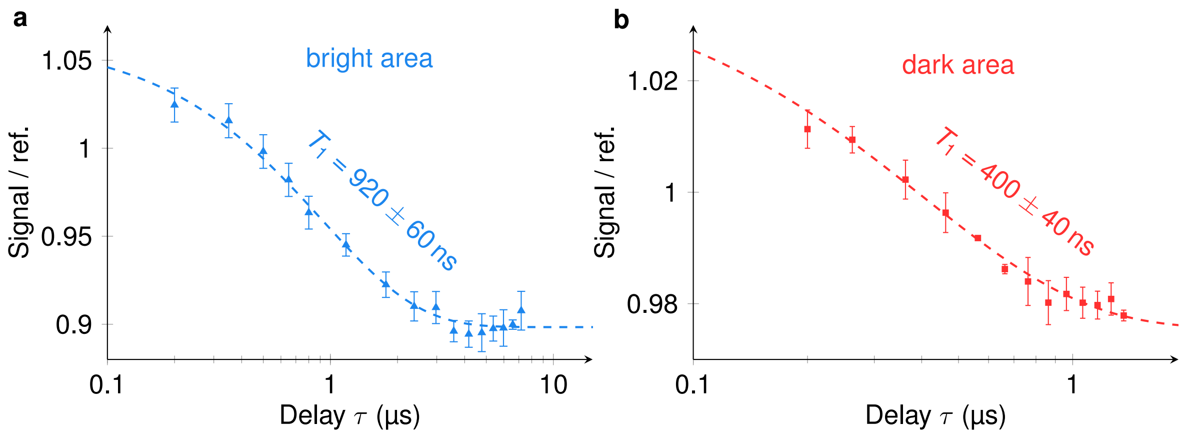

Supplementary Figure 9. T_1 **measurements on the spiral sample.** NV spin relaxation curves measured above **a** the bright and **b** the dark PL areas of the spiral. We observe a reduction of T_1 from $920 \pm 60\,\mathrm{ns}$ to $400 \pm 40\,\mathrm{ns}$.

4.4. Additional relaxation time measurements on the skyrmion sample

We also measured T_1 on the skyrmion sample (Supplementary Fig. 10). We obtain a decrease of T_1 from $9.1 \pm 0.6\,\mu s$ above the background to $870 \pm 60\,ns$ above the skyrmion. The T_1 values are again very small, which can be explained by the dispersion shown in Supplementary Fig. 5. In this sample, the spin wave gap is much smaller than in the domain wall sample and strong modes are located in the NV detection zone in terms of frequency and wavelength.

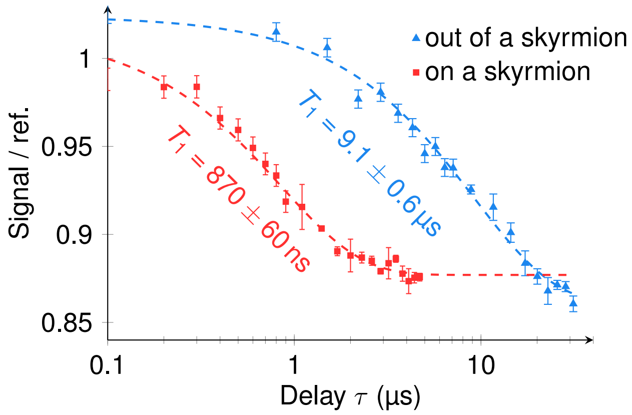

Supplementary Figure 10. T_1 **measurements on the skyrmion sample.** NV spin relaxation curves measured above the background (blue triangles) and above a skyrmion (red squares). We observe a reduction of T_1 when we place the tip above the skyrmion ($T_1 = 870 \pm 60\,ns$) compared the value obtained far from the skyrmion ($T_1 = 9.1 \pm 0.6\,\mu s$).

[1] L. Rondin, J.-P. Tetienne, P. Spinicelli, C. Dal Savio, K. Karrai, G. Dantelle, A. Thiaville, S. Rohart, J.-F. Roch, and V. Jacques, Applied Physics Letters **100**, 153118 (2012).
[2] P. Maletinsky, S. Hong, M. S. Grinolds, B. Hausmann, M. D. Lukin, R. L. Walsworth, M. Loncar, and A. Yacoby, Nature Nanotechnology **7**, 320 (2012).
[3] P. Appel, E. Neu, M. Ganzhorn, A. Barfuss, M. Batzer, M. Gratz, A. Tschope, and P. Maletinsky, Review of Scientific Instruments **87**, 063703 (2016).
[4] J. M. Binder, A. Stark, N. Tomek, J. Scheuer, F. Frank, K. D. Jahnke, C. Müller, S. Schmitt, M. H. Metsch, T. Unden, T. Gehring, A. Huck, U. L. Andersen, L. J. Rogers, and F. Jelezko, SoftwareX **6**, 85 (2017).
[5] T. Hingant, J.-P. Tetienne, L. J. Martínez, K. Garcia, D. Ravelosona, J.-F. Roch, and V. Jacques, Physical Review Applied **4**, 014003 (2015).
[6] A. Dréau, M. Lesik, L. Rondin, P. Spinicelli, O. Arcizet, J.-F. Roch, and V. Jacques, Physical Review B **84**, 195204 (2011).
[7] M. Pelliccione, B. A. Myers, L. M. A. Pascal, A. Das, and A. C. Bleszynski Jayich, Phys. Rev. Applied **2**, 054014 (2014).
[8] D. Schmid-Lorch, T. Häberle, F. Reinhard, A. Zappe, M. Slota, L. Bogani, A. Finkler, and J. Wrachtrup, Nano Lett. **15**, 4942 (2015).
[9] J.-P. Tetienne, A. Lombard, D. A. Simpson, C. Ritchie, J. Lu, P. Mulvaney, and L. C. L. Hollenberg, Nano Lett. **16**, 326 (2016).
[10] J.-P. Tetienne, T. Hingant, L. Rondin, A. Cavaillès, L. Mayer, G. Dantelle, T. Gacoin, J. Wrachtrup, J.-F. Roch, and V. Jacques, Physical Review B **87**, 235436 (2013).
[11] A. Vansteenkiste, J. Leliaert, M. Dvornik, M. Helsen, F. Garcia-Sanchez, and B. Van Waeyenberge, AIP Advances **4**, 107133 (2014).
[12] J. Leliaert, B. Van de Wiele, A. Vansteenkiste, L. Laurson, G. Durin, L. Dupré, and B. Van Waeyenberge, Journal of Applied Physics **115**, 233903 (2014).
[13] C. L. Degen, F. Reinhard, and P. Cappellaro, Reviews of Modern Physics **89**, 035002 (2017).
[14] F. Nörtemann, R. L. Stamps, and R. E. Camley, Physical Review B **47**, 11910 (1993).
[15] W. Brown, Physical Review **130**, 1677 (1963).
[16] J. García-Palacios and F. Lázaro, Physical Review B **58**, 14937 (1998).
[17] J. Leliaert, J. Mulkers, J. De Clercq, A. Coene, M. Dvornik, and B. Van Waeyenberge, AIP Advances **7**, 125010 (2017).
